# Evaluating Patient Preferences and Clinical Outcomes in Stress Urinary Incontinence Treatment: A Short-Term Follow-Up Study of the Transobturator Tape Procedure and Pubourethral Ligament Plication (a Minimally Invasive Technique)

**DOI:** 10.3390/jpm14010034

**Published:** 2023-12-26

**Authors:** Simona Brasoveanu, Răzvan Ilina, Ligia Balulescu, Marilena Pirtea, Cristina Secosan, Dorin Grigoraș, Flavius Olaru, Dragos Erdelean, Oana Balint, Mădălin-Marius Margan, Cristiana-Smaranda Ivan, Laurențiu Pirtea

**Affiliations:** 1 Department of Obstetrics and Gynecology, Victor Babes University of Medicine and Pharmacy, 300041 Timisoara, Romania; simona.brasoveanu@umft.ro (S.B.); ligia.balulescu@umft.ro (L.B.); marilena.pirtea@umft.ro (M.P.); secosan.cristina@umft.ro (C.S.); grigoras.dorin@umft.ro (D.G.); olaru.flavius@umft.ro (F.O.); erdelean.dragos@umft.ro (D.E.); balint.oana@umft.ro (O.B.); pirtea.laurentiu@umft.ro (L.P.); 2 Department of Surgery, Discipline of Surgical Semiology II, Victor Babes University of Medicine and Pharmacy, 300041 Timisoara, Romania; 3 Department of Functional Sciences, Discipline of Public Health, Victor Babes University of Medicine and Pharmacy, 300041 Timisoara, Romania; margan.madalin@umft.ro; 4 General Medicine, Victor Babes University of Medicine and Pharmacy, 300041 Timisoara, Romania; smaranda.ivan@student.umft.ro

**Keywords:** pubourethral ligament, SUI, transobturator tape procedure

## Abstract

Objective: This study aims to provide an in-depth analysis of patient preferences and clinical outcomes associated with two surgical techniques for treating stress urinary incontinence (SUI): the transobturator suburethral sling (TOT) procedure and the pubourethral ligament plication (PUL) procedure. We evaluated the rates of postoperative complications, the duration of each procedure, hemoglobin loss, and days of hospitalization. Materials and Methods: This prospective study included 80 patients who underwent surgery for SUI: 40 patients for the TOT procedure and 40 patients for the PUL procedure. Clinical data on patient characteristics, treatment efficacy, and post-surgical outcomes were analyzed to assess patient preferences and real-world clinical effectiveness. Results: Regarding patient preferences, those who underwent TOT surgery were more likely to be older, had a higher average number of pregnancies, and were more often postmenopausal, in contrast to those who underwent PUL surgery (*p* < 0.001 for each comparison). TOT patients had a hospital stay on average of 1.02 days, while PUL patients benefited from ambulatory stays only. In addition, the TOT group had a significantly longer average operating time (16.80 min) compared to the PUL group (9.90 min, *p* < 0.001). The study revealed notable outcomes in both groups, with high cure rates for both TOT (N_1_ = 33, 82.5%) and PUL (N_2_ = 28, 70%) procedures. Specifically, 76.25% of the patients (61 out of 80) were cured after the procedures. Chronic pelvic pain was present in 3.75% of all patients and was notably only observed in the TOT group, with 3 (7.5%) cases being noted. Similarly, vaginal erosion was experienced by 5% of all patients, with 10% of patients in the TOT group and none in the PUL group being affected. Dyspareunia occurred in 2.5% of all patients, with there being two (5%) cases in the TOT group and none in the PUL group. Conclusions: This study highlights that while the PUL procedure achieves cure rates comparable to TOT, it offers a less invasive option with shorter operating times and no hospitalization required. These findings suggest that PUL could be a viable alternative for stress urinary incontinence (SUI) treatment, especially in contexts where avoiding mesh use is preferred. This adds significant value to patient-centered care in SUI management, offering tailored treatment options based on patient characteristics, preferences, and risk profiles.

## 1. Introduction

The estimated prevalence of SUI in women becomes more frequent as they age (ranging from 14.8% to 31.8% in women over 50 years of age), with its prevalence rising during early adulthood [1]. Approximately 4% of women are anticipated to undergo surgical intervention for urinary incontinence [2]. Projections suggest that by 2050, due to increased life expectancy and the notable expansion of the adult population in developed nations, as many as 28.4 million women in the United States could potentially be affected by urinary incontinence [3].

The field of surgical treatment for SUI has undergone substantial evolution over the past two decades, with each procedure designed to address the limitations of previous procedures, and today, more than 120 different surgical procedures have been tried for SUI in females [4,5].

The primary surgical procedure frequently used to treat SUI in women involves mesh placement. This technique witnessed approximately 3.7 million mesh units sold worldwide between 2005 and 2013 with an estimated 10,000,000 operations to date. In the United States, the FDA has introduced a proposal to increase the risk classification of urogynaecological meshes, requiring premarket notification and the enforcement of specific controls [6].

Numerous case series have indicated that SUI procedures involving meshes can result in chronic pain, urethral fistula, substantial voiding difficulties, and mesh erosion into the urethra or vagina [7].

The increasing global debate surrounding vaginal meshes has triggered legal action against manufacturers on a global scale, leading to the withdrawal of certain products. Considering this situation, patients are requesting alternative treatments to cure SUI without tapes and for them to be effective and with minimal or without complications.

Meshes gained interest after the FDA warnings, and several procedures such as the use of peri-urethral bulking agents have been proposed [8]. Another type of surgery is retropubic suspension procedures such as open or laparoscopic Burch colposuspension. In this type of procedure, the fundamental principle is to elevate and secure the bladder neck and the proximal urethra in a retrograde position to provide improved support [9].

Transobturator tape is a synthetic tape specifically crafted for urethral suspension in the management of female stress urinary incontinence (SUI). Notably, this tape possesses two distinctive characteristics: its non-woven polypropylene composition is overlaid with silicone on the urethral side, thereby restricting polypropylene retraction and creating a barrier against the expansion of periurethral fibrosis. The method of transmuscular insertion, involving the obturator and puborectalis muscles, mimics the natural suspension fascia of the urethra, all the while safeguarding the retropubic space [10]. 

Going back to Integral Theory by Petros, stress and urge symptoms might both originate from a common anatomical issue, specifically a lax vagina, although for distinct reasons. This laxity could stem from deficiencies within the vaginal wall or its supportive structures, such as ligaments, muscles, and connective tissue attachments [4].

In 1961, Robert Zacharin [11] described the pubourethral ligament (PUL) and the vaginal attachment of the levator ani. He postulated that these anatomical structures played a significant role in urinary continence but without any information on the mechanism. Furthermore, there are mentions of pubourethral ligaments in various sources (Curtis, Anson, and McVay, 1939; Krantz, 1951; Irvine, 1956; Langrader, 1956). However, a comprehensive description of all of the components contributing to the suspension of the entire urethral length has not been provided. These authors acknowledged the significance of pubourethral ligaments as crucial elements in urethral support, with Langrader emphasizing the remarkable strength of these ligaments [11].

Only two studies presented their results after the PUL procedure was performed. Sivaslioglu et al. reported a cure rate of 86%, 31/36 women, and five surgical failures, four immediately post-operatively and one at 3 months [12]. Petros et al. documented an absence of cough-induced urinary leakage in 30 out of 31 women during the postoperative evaluation, a finding that was promising yet not definitive [13].

Objective: The primary objective of this study is to provide a comprehensive evaluation of patient preferences and clinical outcomes in the treatment of stress urinary Incontinence (SUI), focusing on the transobturator suburethral sling (TOT) procedure and the pubourethral ligament plication (PUL) procedure. The study aims to assess the efficacy, safety, and patient satisfaction associated with these techniques, while also evaluating key perioperative parameters such as surgery duration, hemoglobin loss, and length of hospital stay. This research seeks to contribute to the understanding of optimal treatment approaches in SUI, enhancing patient-centered care in this field.

## 2. Materials and Methods

### 2.1. Patient Selection and Inclusion Criteria

We conducted a prospective cohort analysis encompassing all eligible participants (80 patients) who underwent surgery for stress urinary incontinence (SUI) at the Department of Obstetrics and Gynecology of Timisoara University City Hospital, spanning from January 2019 to December 2020. The cohort consisted of 80 patients, with 40 undergoing the transobturator tape (TOT) procedure and 40 undegoing the pubourethral ligament (PUL) procedure. The study was carried out following approval from the Human Ethical Committee of the University of Medicine and Pharmacy “Victor Babes,” Timisoara, Romania (approval number: 63/17 December 2018) in accordance with ethical standards. All interventions conducted in the study involving human participants adhered to the principles outlined in the Helsinki Declaration (as revised in 2013). Written informed consent was obtained from each patient.

Patients opted for either the TOT procedure or the PUL procedure based on thorough discussions with their doctor. These discussions thoroughly explored potential risks, complications, and literature-reported cure rates for each procedure, enabling a more informed and collaborative decision-making process.

In continuation, we conducted a comprehensive clinical evaluation of each patient. This evaluation included a standardized history, urogynecology examination, cough test, and urinalysis. 

Women experiencing SUI may also encounter issues related to urination, such as overactive bladder, dysfunctional voiding, or detrusor underactivity. Additionally, some patients may exhibit an elevated post-void residual volume before surgery, potentially impacting the treatment results. 

The study enrolled adult female patients, aged 18 years or older, who were clinically diagnosed with authentic stress urinary incontinence (SUI) and exhibiting symptoms. These patients had normal urethral closing pressure and demonstrated a positive result on the cough test. As a requisite element of the study’s protocol, all participants were required to fill out the Urinary Distress Inventory-6 (UDI-6) questionnaire, which was administered both during their initial evaluation and at subsequent follow-up appointments.

Exclusion criteria included patients with mixed urinary incontinence and certain comorbidities. More precisely, we excluded female patients who had urinary incontinence characterized by significant urgency and urge urinary incontinence diagnosed using a bladder diary. Additionally, we excluded those with stress urinary incontinence (SUI) attributed to low urethral closing pressure. Patients with certain specific conditions that had the potential to influence the study results were also excluded. These comorbidities encompass uterovaginal descent greater than grade 1, cystocele, urinary tract infections that were unresponsive to treatment, patients undergoing antipsychotic treatment (due to the possibility of developing urinary retention during such treatment), and those who were currently pregnant. Patients with a history of prior vaginal repair or recurrent incontinence were likewise not included in our study. 

Both procedures were performed by the same surgical team. A single dose of an antibiotic for prophylaxis was administrated preoperatively. The Foley catheter was removed 12–24 h after surgery in the TOT group and no Foley catheter was placed in the PUL group.

The following parameters were evaluated for each patient: the duration of surgery, hospital stay, hemoglobin loss, and postoperative complications, including the results of the validated UDI-6 questionnaire completed both before surgery and at follow-up visits.

The follow-up period comprised evaluation at 6, 12, and 18 months after the procedure. The follow-up visits targeted the following parameters: the cure rate, the rate of significant improvement, and complications: acute urinary retention, de novo impetuosity, dyspareunia, chronic pelvic pain, and mesh erosion. The surgeon carried out a clinical examination that involved sling palpation in the TOT group, checking the vaginal mucosa for mesh erosion, and performing a cough stress test.

Outcomes: The cure rate was defined by the absence of leakage during the pad test and/or by a negative stress maneuver (cough test or Valsalva test). Other outcomes included the duration of surgery, hospital stay, hemoglobin loss, de novo impetuosity, acute urinary retention, dyspareunia, chronic pelvic pain, and vaginal erosion.

#### 2.1.1. The Surgical Technique Description for PUL

The procedure is performed under intravenous anesthesia. The patient is positioned in the lithotomy position, with the hip hyperflexed.

**Figure 1:**
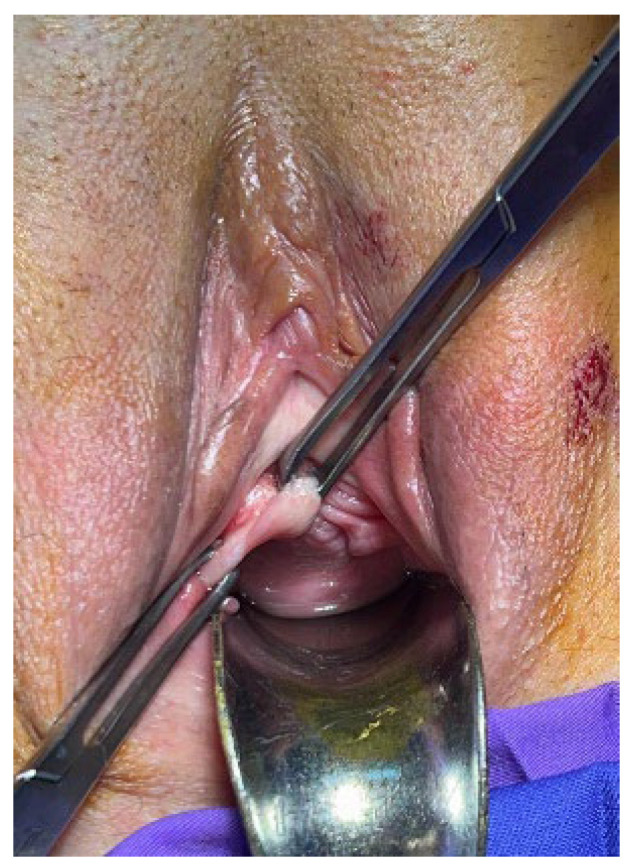
Vaginal mucosa between two grasping forceps under tension.

**Figure 2:**
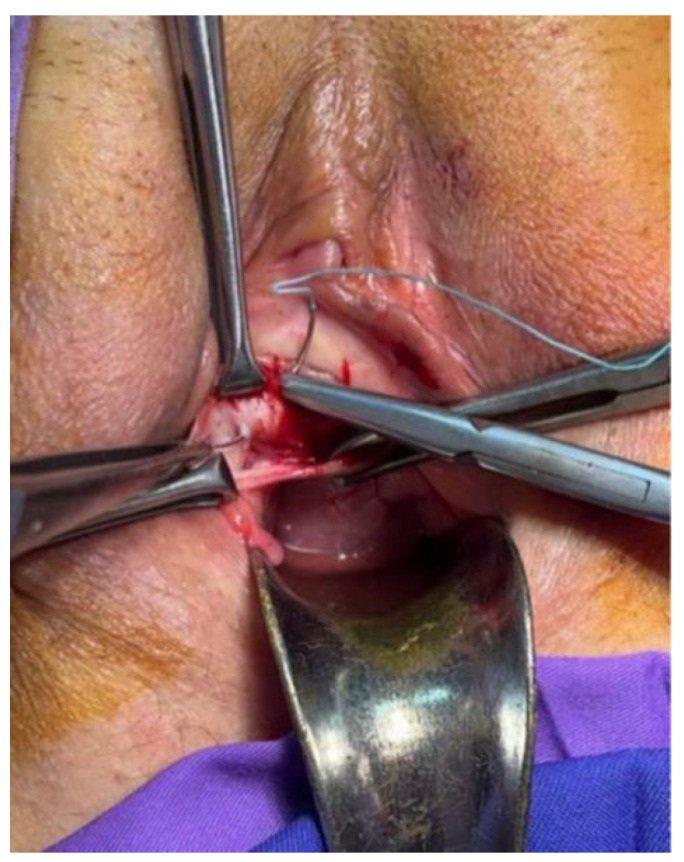
The 3.0 polyester suture penetrates the PUL.

**Figure 3:**
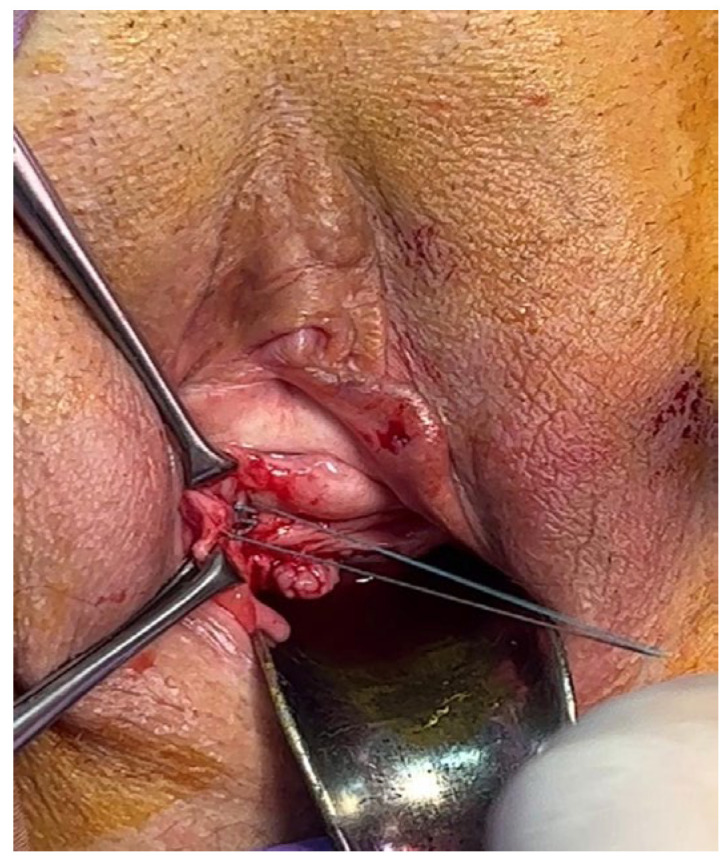
Aspect of the suture before it is tied.

Two 2 cm oblique incisions of vaginal mucosa are made in each sulcus, between two grasping forceps, under tension, from the bladder neck to the pubic bone (Figure 1). With dissecting scissors, the incisions are opened out laterally to reveal the two branches of the PUL. A No.3.0 polyester suture penetrates the PUL (Figure 2), the proximal part of the pubococcygeus muscle (PCM), and the fascial tissues behind the pubic bone. The sutures are tied just sufficiently to bind the structures together (Figure 3) to prevent PUL extension, which causes SUI. The vaginal mucosa incisions are sutured with 2.0 Vicryl.

The TOT procedure was performed according to the technique developed by Delorme [10].

#### 2.1.2. Technique Description

The standard transobturator tape (TOT) procedure is conducted under spinal anesthesia. The patient assumes the lithotomy position, with the hip in hyperflexion. A 2 cm incision is made on the anterior vaginal wall, specifically over the midurethra. Blunt dissection extends laterally until the index finger reaches the inner surface of the ischiopubic bone and obturator foramen. Bilateral horizontal lines are drawn from the level of the clitoris to the inguinofemoral sulcus. A 1 cm vertical skin incision is made at the point where these lines intersect the sulcus. Specially designed needles are used to perforate the obturator membrane, and then the needle is turned medially. It is guided with a finger in the vaginal incision to exit in the vagina. The tape is loaded onto the needle and pulled through the skin incision on each side. Tension is adjusted to allow dissecting scissors to lie flat easily between the tape and the urethra. The incisions are closed using 2.0 Vicryl sutures. The Foley catheter is removed 12–24 h after surgery.

### 2.2. Statistical Analysis

Data were gathered through the Mediflux software platform (v1.1, Origini Health™, Boston, MA, USA). 

Statistical assessments were executed using Python 3.9.13 (Python Software Foundation™, Wilmington, DE, USA). The Pandas library facilitated data handling, while the SciPy library managed statistical calculations.

Descriptive statistics highlighted the demographic and clinical attributes of the patient cohorts. Continuous variables were represented by means and standard deviations (SD), whereas categorical variables were illustrated with counts and percentages. The Shapiro–Wilk test was utilized to confirm normality assumptions. The Mann–Whitney U test was mainly used to compare continuous and ordinal variable means between the two patient groups. In cases where the distribution was normal, the *t*-test was applied.

Regarding categorical variables, the Chi-squared test was primarily used in instances with satisfactory expected frequencies in the contingency table cells. However, due to limited sample sizes, Fisher’s exact test was employed when necessary.

Across all analytical methods, a *p*-value below 0.05 was considered to indicate statistical significance.

## 3. Results

A total of 80 eligible patients meeting the inclusion criteria were enrolled in our study, with 40 patients (50%) choosing the “TOT” technique and an equal number of 40 patients (50%) opting for the “PUL” technique.

The demographic and clinical characteristics of the 80 enrolled patients are outlined in Table 1. The patients were categorized into two groups based on the performed surgical intervention: 40 (50%) in the “TOT” group and 40 (50%) in the “PUL” group.

Significant disparities were noted in age, parity, and postmenopausal status between the two groups. The mean age of patients in the TOT group was 63.80 years, surpassing the mean age of 51.42 years in the PUL group (*p* = 0.001). The mean body mass index (BMI) for the TOT group was 28.73, marginally higher than the PUL group’s mean BMI of 27.99 (*p* = 0.182).

Concerning menopausal status, there was a markedly higher percentage of postmenopausal women in the TOT group (100%) in contrast to the PUL group (30%) (*p* < 0.001). Additionally, patients in the TOT group exhibited a higher mean parity of 2.20, compared to a mean parity of 0.98 in the PUL group (*p* = 0.002).

In summary, patients who underwent TOT surgery were noticeably older, had a higher mean parity, and were more frequently postmenopausal compared to those who underwent PUL surgery.

Table 2 provides a summary of the perioperative outcomes for the two surgical procedures.

Hemoglobin loss, indicating the change in hemoglobin levels before and after surgery, was determined to be 0.96 for the entire study group. When examining the subgroups, the TOT group showed a hemoglobin loss of 0.99, while the PUL group showed a hemoglobin loss of 0.93. Importantly, there was no statistically significant difference between these two subgroups (*p* = 0.381).

In terms of average hospital stay, the TOT group had a mean hospital stay of 1.02 days (SD: 0.15). The PUL group was composed of patients who were not hospitalized, as they had just an ambulatory stay, as per the protocol of the procedure.

Finally, the average duration of the surgical procedures was evaluated for both operative techniques. For the entire study group, the mean operating time was 13.35 min (SD: 4.10). When the techniques were analyzed separately, the TOT group had a notably longer operating time, averaging 16.80 min (SD: 2.76), compared to the PUL group, which had an average operating time of 9.90 min (SD: 1.46). This difference was highly statistically significant (*p* < 0.001).

The analysis of postoperative complications within the study groups is presented in Table 3. Among the entire study cohort, 1.25% encountered DN imperiosity, with one case (2.5%) observed in the TOT group and none observed in the PUL group. Likewise, acute urinary retention occurred in 1.25% of the overall study group, with only one case (2.5%) identified in the TOT group.

Chronic pelvic pain was present in 3.75% of all patients and was notably only in the TOT group, with 3 cases (7.5%) being noted. Similarly, vaginal erosion was experienced by 5% of patients, with 10% in the TOT group and none in the PUL group.

Dyspareunia occurred in 2.5% of all patients, with only 2 cases (5%) being reported in the TOT group.

In summary, the PUL group had no complications reported. These results indicate that both surgical procedures have low postoperative complication rates, but pubourethral ligament plication seems to be the safer choice. 

The findings related to the results of the surgical interventions are detailed in Table 4.

In both groups, the TOT group (33 cases, 82.5%) and the PUL group (28 cases, 70%), the cure rates were notably high. Specifically, after the procedures, 76.25% of the patients (61 out of 80) experienced a cure. However, it is worth noting that this disparity in cure rates did not yield statistical significance (*p* = 0.293). The postoperative improvement rate was significantly enhanced, with 12.5% (10 out of 80) of patients showing better outcomes, split into 10% in the TOT group (4 cases) and 15% in the PUL group (6 cases).

At the 6-month follow-up, both groups, the TOT group (30 cases, 75%) and the PUL group (26 cases, 65%) maintained high cure rates. Specifically, 70% of the patients (56 out of 80) were cured after the procedures. However, the difference in cure rates was not statistically significant (*p* = 0.464). The improvement rate at the 6-month follow-up was also comparable between the two groups, with this being 10% in the TOT group (4 cases) and 7.5% in the PUL group (3 cases).

At the 12-month follow-up, high cure rates persisted in both groups, with this being presented in the TOT group (29 cases, 72.5%) and the PUL group (24 cases, 60%). Specifically, 66.25% of the patients (53 out of 80) were cured after the procedures. However, once again, the difference in cure rates did not achieve statistical significance (*p* = 0.344). Likewise, the improvement rate at the 12-month follow-up remained similar in both groups, with this being 10% in the TOT group (four cases) and 5% in the PUL group (two cases). Notably, this difference in cure rates was also not statistically significant (*p* = 0.675).

Lastly, at the 18-month follow-up, high cure rates were observed in both groups, with this being presented in the TOT group (26 cases, 67.5%) and the PUL group (24 cases, 60%). Specifically, 63.75% of the patients (51 out of 80) were cured after the procedures. However, as in previous intervals, the difference in cure rates did not reach statistical significance (*p* = 0.641). The rate of significant improvement at the 18-month follow-up was comparable between the two groups, with this being 10% in the TOT group (four cases) and 5% in the PUL group (two cases). Again, this difference in cure rates was not statistically significant (*p* = 0.675).

In the univariate logistic regression analysis, predictor variables such as age, post-menopausal status, and the selected surgical procedure were assessed and had no significant impact on the chances of treatment failure (defined as not cured) in the entire study group and both the TOT and PUL groups (*p* > 0.05 for all variables). Only parity was found to adversely influence the failure rate, consequently predicting the actual success rate in the study group and TOT group, but not in the PUL group (study group: logit(*p*) = −0.0030 + −0.6687 × parity, *p* = 0.018; TOT group: logit(*p*) = 0.9929 + −1.2877 × parity, *p* = 0.019). 

The validated Urinary Distress Inventory-6 (UDI-6) questionnaire was completed before surgery and at a follow-up visit. The scores are presented in Table 5. 

The average UDI-6 score before the procedure for the entire study group stood at 41.30 (SD: 25.72). In the TOT group, the mean preoperative UDI-6 score was slightly higher at 42.07 (SD: 25.53), while the PUL group displayed a mean preoperative UDI-6 score of 40.54 (SD: 26.21), with no statistically significant distinction between the two groups (*p* = 0.775)

After the procedure, the average UDI-6 score for the entire study group was 12.40 (SD: 6.47). Within the TOT group, the mean postoperative UDI-6 score decreased to 10.31 (SD: 4.80), whereas the PUL group exhibited a slightly higher mean postoperative UDI-6 score of 14.23 (SD: 7.30), again with no statistically significant difference between the two groups (*p* = 0.146).

## 4. Discussion

According to Petros’s integral theory, a lax vagina is a common anatomical defect that can cause SUI and urge symptoms. This laxity can appear because of defects within the vaginal wall itself or in its supporting tissues, such as muscles, ligaments, or connective tissue insertions [4]. 

Understanding that the vagina has two separate anatomical segments that are pulled in opposite directions against the pubourethral ligament, which serves as a fulcrum, is crucial to understand this theory. The zone of critical elasticity of the vagina must be elastic enough to transfer these movements. Laxity may be brought on by altered collagen/elastin in the vaginal connective tissue and/or its ligamentous supports [4]. 

The resilience of the pubourethral ligament is rooted in its connective tissue constituents. Connective tissue undergoes degeneration with the aging process and childbirth. Analogous to a sail with lax attachments fluttering in the breeze, the pubourethral ligament, along with hammock laxity, may impede the impact of muscle forces on urethral closure. Among these components, the pubourethral ligament holds particular significance, serving as an anchor for the muscle forces that elongate and constrict the urethra during exertion [4].

To mitigate pubourethral ligament extension, assuming the validity of assumptions regarding neocollagen formation derived from calculations in the primary author’s Doctor of Surgery thesis, No. 5 or even No. 2 polyester sutures are employed. These sutures secure the pubourethral ligament to connective tissue structures beneath the symphysis, vagina, and pubococcygeal muscle laterally. This approach is intended to be adequate for repairing the weakened ligament, given the anticipated formation of neocollagen [4,14].

In terms of cure rate, in our study, there was no significant difference between the two groups. Specifically, 76.25% of the patients (61 out of 80) were cured after the procedures, with 33 patients cured in the TOT group and 28 cured in the PUL group. Bandarian et al. documented a complete cure rate of 90.3% in the TOT group [15]. In a prospective trial conducted by Sivaslioglu et al. [16], both subjective and objective cure rates for SUI at the one-year mark stood at 85.7% within the TOT group. Additionally, at the two-year follow-up, the subjective and objective cure rates remained similar, at 87.5% within the TOT group (32 out of 49 patients were available for assessment). Ascioglu et al., in a study involving 272 patients, reported an objective cure rate of 77.5% and a subjective cure rate of 81.7% in the TOT group, with a follow-up period extending to five years [17].

Petros et al. reported in their study negative cough tests on 30/31 women before patient discharge [13] for patients who underwent the PUL procedure, and Sivaslioglu et al. reported a cure rate of 86%, 31/36 women, and five surgical failures, four immediately post-operatively and one at 3 months after the PUL procedure [12].

Since the 1960s, polypropylene (PP) implants have been utilized to treat inguinal and ventral hernia, and since the 1990s, POP (elvic organ prolapse) and SUI have both been treated with them. Although it has been demonstrated that PP implants reduce the chance of recurrence, the advantages must be evaluated against the dangers associated with mesh-related complications [6].

Regarding postoperative complications, there were no significant differences between the two groups in terms of acute urinary retention and de novo urgency. However, a notable difference was found in the occurrence of vaginal erosion, with four cases (10%) reported in the TOT group compared to none in the PUL group. Additionally, chronic pelvic pain was documented in 3 cases (7.5%) within the TOT group.

In Lukban’s study on TOT placement, 6% (out of 33 patients) indicated a decreased ability to engage in sexual relationships. Furthermore, 14.9% of the patients reported experiencing vaginal pain, pressure, or protrusion [18]. In a cohort of 233 women who underwent TOT, followed up for 27 months, de novo dyspareunia was observed in 9% of patients [19].

Mesh exposure and extrusion, stemming from the utilization of a synthetic polypropylene mesh, constitute the primary and highly debated complications associated with mesh implantation. The incidence of this complication is anticipated to exceed 12%, as the risks of mesh exposure and extrusion escalate over time, posing challenges for removal in the presence of long-term complications arising from polypropylene integration with the tissue. A considerable number of patients may contend with persistent pain, reporting issues such as dyspareunia, vaginal bleeding, and vaginal discomfort [3]. It is estimated that approximately 9.8% of patients undergoing surgical mesh insertion for stress urinary incontinence (SUI) are prone to developing complications either peri-procedurally, within 30 days, or over a five-year span [4,20].

Kokanali et al. conducted a multivariate analysis, concluding that mesh erosion was independently linked to various risk factors. These factors included advanced age, the presence of diabetes mellitus, current smoking habits, a vaginal incision exceeding 2 cm in length, and multiple vaginal incisions necessitated by postoperative issues, as well as a history of previous surgeries for pelvic organ prolapse or incontinence [21,22].

Sivaslioglu et al. and Bandarian et al. reported similar results, with the mean hospital stay in the TOT group being 2.06 ± 1.03 days and operation time for the TOT procedure being 20 (15–25) min [15,16].

The PUL procedure is an available procedure, inexpensive, and simple, with less surgical skill required. The procedure is ambulatory surgery, performed under intravenous anesthesia. The procedure is considerably less invasive as it does not involve any devices that could potentially damage the bladder, nerves, blood vessels, or bowel. Additionally, there are no tapes used that might lead to issues like postoperative urinary retention or urethral perforations.

To our knowledge, only two studies presented their results after the PUL procedure [12,13].

The study is limited by a small sample size (80 patients) and a single-center setting, potentially restricting its applicability to a wider population and different healthcare settings. It focuses on specific patient characteristics, has a short follow-up period (6–18 months post-procedure), and relies on self-reported data, which may introduce bias. The study also lacks a predefined sample size calculation, shaped by its patient-centered approach, practical clinical constraints, and its exploratory nature. These limitations suggest caution when applying the study’s findings to the treatment of SUI, although offering a non-mesh alternative (PUL) for SUI treatment may be a valid choice. Nonetheless, it offers valuable preliminary insights into patient preferences and outcomes in stress urinary incontinence treatment, serving as a foundation for more comprehensive future research.

## 5. Conclusions

In summary, our study suggests that the pubourethral ligament plication (PUL) procedure yields cure rates comparable to those of the transobturator sling (TOT) procedure, offering a potential mesh-free alternative for treating stress urinary incontinence (SUI). This is particularly significant considering mesh restrictions in certain countries and the ongoing exploration of non-mesh options. 

Nonetheless, it is crucial to recognize that our study featured a relatively short follow-up period, limiting our ability to thoroughly assess long-term comparative outcomes’ further research is essential to validate and expand upon these initial findings.

## Figures and Tables

**Table 1:** Clinical characteristics of the patients.

Parameter	Mean (95%CI) or N (%)			
	Study Group N = 80	TOT Group N_1_ = 40 (50%)	PUL Group N_2_ = 40 (50%)	*p*-Value
Age	57.61 (55.43–59.79)	63.80 (61.65–65.95)	51.42 (48.76–54.09)	0.001 ^1,^*
BMI	28.36 (27.95–28.77)	28.73 (28.36–29.10)	27.99 (27.28–28.71)	0.182 ^1^
Parity	1.88 (1.68–2.10)	2.20 (1.95–2.45)	0.98 (1.27–1.88)	0.002 ^1,^*
Postmenopausal	52 (65%)	40 (100%)	12 (30%)	<0.001 ^2,^*

^1^ Mann–Whitney U test *p*-value; ^2^ Chi-square test *p*-value; * statistically significant.

**Table 2:** Perioperative outcomes of the surgical techniques.

Outcome	Mean (SD)			
	Study Group N = 80	TOT Group N_1_ = 40 (50%)	PUL Group N_2_ = 40 (50%)	*p*-Value
Hemoglobin loss (g/dL)	0.96 (0.32)	0.99 (0.31)	0.93 (0.33)	0.381 ^1^
Mean hospital stay (days)	-	1.02 (0.15)	Ambulatory stay	-
Operating time (min)	13.35 (4.10)	16.80 (2.76)	9.90 (1.46)	<0.001 ^2,^*

^1^ *t* test *p*-value; ^2^ Mann–Whitney U test *p*-value; * statistically significant.

**Table 3:** Complications of the surgical procedures.

Postoperative Complications	N (%)		
	Study Group N = 80	TOT N_1_ = 40 (50%)	PUL N_2_ = 40 (50%)
DN imperiosity	1 (1.25%)	1 (2.5%)	0 (0%)
Acute urinary retention	1 (1.25%)	1 (2.5%)	0 (0%)
Dyspareunia	2 (2.5%)	2 (5%)	0 (0%)
Vaginal erosion	4 (5%)	4 (10%)	0 (0%)
Chronic pelvic pain	3 (3.75%)	3 (7.5%)	0 (0%)

**Table 4:** Outcomes of the surgical procedures.

Outcome	N (%)			
	Study Group N = 80	TOT N_1_ = 40 (50%)	PUL N_2_ = 40 (50%)	*p*-Value
Cured				
post-operatively	61 (76.25%)	33 (82.5%)	28 (70%)	0.293 ^1^
at 6-months follow-up	56 (70%)	30 (75%)	26 (65%)	0.464 ^1^
at 12-month follow-up	53 (66.25%)	29 (72.5%)	24 (60%)	0.344 ^1^
at 18-month follow-up	51 (63.75%)	26 (67.5%)	24 (60%)	0.641 ^1^
Significantly improved				
post-operatively	10 (12.5%)	4 (10%)	6 (15%)	0.737 ^2^
at 6-month follow-up	7 (8.75%)	4 (10%)	3 (7.5%)	1.000 ^2^
at 12-month follow-up	6 (7.5%)	4 (10%)	2 (5%)	0.675 ^2^
at 18-month follow-up	6 (7.5%)	4 (10%)	2 (5%)	0.675 ^2^

^1^ Chi-squared test; ^2^ Fischer’s exact test *p*-value.

**Table 5:** Preoperative and postoperative UDI-6 questionnaire scores in the study group.

Parameter	Mean (SD)			
	Study Group N = 80	TOT Group N_1_ = 40 (50%)	PUL Group N_2_ = 40 (50%)	*p*-Value
UDI-6 Preoperative	41.30 (25.72)	42.07 (25.53)	40.54 (26.21)	0.775 ^1^
UDI-6 Postoperative	12.40 (6.47)	10.31 (4.80)	14.23 (7.30)	0.146 ^1^

^1^ Mann–Whitney U test *p*-value.

## Data Availability

Data are contained within the article.
